# Ultrastaging Using Ex Vivo Sentinel Lymph Node Mapping and One-Step Nucleic Acid Amplification (OSNA) in Gastric Cancer: Experiences of a European Center

**DOI:** 10.3390/cancers13112683

**Published:** 2021-05-29

**Authors:** Bruno Märkl, Bianca Grosser, Kerstin Bauer, Dmytro Vlasenko, Gerhard Schenkirsch, Andreas Probst, Bernadette Kriening

**Affiliations:** 1 Institute of Pathology and Molecular Diagnostics, University Hospital Augsburg, 86156 Augsburg, Germany; bianca.grosser@uk-augsburg.de (B.G.); Kerstin.Bauer@uk-augsburg.de (K.B.); 2 Department of Visceral Surgery, University Hospital Augsburg, 86156 Augsburg, Germany; Dmytro.Vlasenko@uk-augsburg.de (D.V.); bernadette.kriening@uk-augsburg.de (B.K.); 3 Tumor Data Management, University Hospital Augsburg, 86156 Augsburg, Germany; Gerhard.Schenkirsch@uk-augsburg.de; 4 Medicine III—Gastroenterology, University Hospital of Augsburg, 86156 Augsburg, Germany; andreas.probst@uk-augsburg.de

**Keywords:** gastric cancer, lymph node staging, histology, OSNA, one-step nucleic acid amplification

## Abstract

**Simple Summary:**

In this study, the effectiveness of One-step nucleic acid amplification (OSNA) in combination with ex vivo SLN mapping is compared with conventional histology including immunohistochemistry. OSNA lymph node evaluation has been performed in 41 gastric cancer cases. It showed a high effectiveness with sensitivity, specificity, and accuracy rates of 85.4%, 93.5%, and 92.4%, respectively The LN status could be predicted in 40 cases and led to upstaging in three cases (14%). The OSNA method proved its potential to increase the sensitivity of metastases detection.

**Abstract:**

Background: In this study, the effectiveness of One-step nucleic acid amplification (OSNA) in combination with ex vivo SLN mapping is compared with conventional histology including immunohistochemistry. Methods: LNs were retrieved from gastrectomy specimens in an unfixed state. After ex vivo SLN mapping using methylene-blue, LNs were sliced to provide samples for histology and OSNA. Results: In total, 334 LNs were retrieved in the fresh state from 41 patients. SLN detection was intended in 40 cases but was successful in only 29, with a correct LN status prediction in 23 cases (79%). Excluding one case out of 41 with a failure likely caused by a processing error, OSNA showed a high effectiveness with sensitivity, specificity, and accuracy rates of 85.4%, 93.5%, and 92.4%, respectively. The LN status could be predicted in all but one case, in which the single positive LN was not eligible for OSNA testing. Moreover, OSNA evaluation led to upstaging from N0 to N+ in three cases (14%). Conclusion: The ex vivo SLN protocol used resulted in a relatively poor detection rate. However, the OSNA method was not hampered by this detection rate and proved its potential to increase the sensitivity of metastases detection.

## 1. Introduction

Gastric carcinoma is a heterogeneous tumor entity with poor prognosis, and the overall survival of patients is limited despite the identification of specific and some environmental risk factors, improved screening measures, and therapeutic strategies. It has the third-highest mortality rate after lung and colon cancer, and with an incidence of approximately 1 million new cases in 2018, it is the sixth most common cancer worldwide [1]. Despite an established molecular classification [2,3] and the still-growing importance of biomarker-based personalized treatment approaches [4], the TNM stage is still of crucial importance for prognosis estimation and therapeutic strategies in gastric cancer [5]. Lymph nodes (LNs) play an essential role in terms of both therapeutic and prognostic outcomes [6].

Prior to the publication of the long-term Dutch D1D2 trial [7], the importance of D2 lymphadenectomy was debated in the Western world. The D2 approach is now widely accepted as the recommended therapy in resectable advanced gastric cancers by the leading national and international associations. Nevertheless, the amount of total gastrectomy and D2 lymphadenectomy could be reduced if more limited procedures with identical oncological safety would be available. The concept of sentinel lymph node (SLN) mapping ideally combined with intra-operative SLN evaluation potentially represents such an approach [8], especially if used in combination with local resection methods like endoscopic submucosal dissection (ESD) [9].

Unfortunately, the conventional intra-operative frozen section analysis of SLNs is hampered by a high rate of false-negative results, making its value in this specific situation questionable [10]. Molecular techniques are promising alternatives or adjuncts to conventional histological techniques. This is also the case in terms of ultrastaging, which addresses the issue of undetected small metastases or isolated tumor cells. The role of micro-metastases in gastric cancer, however, remains unclear [11]. In terms of micro-metastases, a meta-analysis revealed that only molecular LN staging was a useful prognostic technique for colon cancer [12].

One-step nucleic acid amplification (OSNA) is a technique for evaluating LN metastases based on the detection of the mRNA of cytokeratin 19 (CK19). It was initially developed for intra-operative SLN evaluation in breast cancer [13], and its suitability was demonstrated in a meta-analysis of 19 studies with over 6000 LNs [14]. It can evaluate LNs within 30 min, and its feasibility in several other cancer entities, including lung, thyroid, uterus, head and neck, and prostate cancers, has been demonstrated previously [15,16,17,18,19]. Another focus was on tumors of the gastrointestinal tract, mainly on colorectal cancers [20]. Conducting a multicenter study, Itabashy et al. could demonstrate that OSNA is not only safe but moreover prognostic in stage II colon cancer even after multivariable analysis [21]. In addition, some studies addressing OSNA have also been conducted with gastric carcinoma [22], with two of them specifically evaluating SLNs [23,24].

Most of the OSNA-related studies have been performed in Asia [13]. This study aimed to evaluate the technique in a European center, where dissecting LNs in the fresh state is not part of the routine pathology procedures. A particular focus was placed on its potential to improve LN staging in gastric cancer by detecting metastases with increased sensitivity. The evaluation of its feasibility in combination with an ex vivo SLN mapping technique was the secondary study aim. It is noteworthy that the rationale behind this study’s sentinel mapping concept differs fundamentally from the other published studies combining SLN mapping with OSNA [23,25]. Usually, SLN mapping aims to reduce the extent of an operation. Ex vivo SLN mapping, used in this study, was performed to apply sophisticated and expensive techniques in a focused manner on LNs that have the highest probability of carrying metastases. This concept is applied in the field of colorectal cancer more than any other [26].

## 2. Materials and Methods

### 2.1. Study Cohort

Inclusion criteria were gastrectomy due to confirmed adenocarcinoma of the stomach or the gastroesophageal junction of all stages regardless of the administration of neoadjuvant chemotherapy. Completion resections after initial local resections were also included. Other entities besides adenocarcinoma were excluded. To measure the effect of a potential learning curve effect regarding the lymph node dissection, the study was divided into a first (cases 1–20) and a second period (cases 21–42).

From February 2016 to September 2017, 42 cases of gastric cancer were prospectively and consecutively enrolled. Case 2 had to be excluded because no LN could be detected in the fresh specimen. The clinicopathological characteristics of the remaining 41 cases are summarized in Table 1. Informed and written consent was obtained from all patients. The study was approved by the internal review board of the Klinikum Augsburg (No. 2015-02). Follow-up data were provided by the Tumor Registry of the University Clinic Augsburg and by screening the clinic information system and the files provided by the Department of Pathology and Molecular Diagnostics.

### 2.2. Specimen Preparation and Ex Vivo SLN Mapping

All specimens were brought to the pathology department immediately after resection without time delay and placed on crushed ice after washing. The resection margins were sampled for an intraoperative evaluation on the frozen sections. After that, the specimens were investigated palpatory to identify the tumor region. Then, approximately 1 mL of unsterilized Löffler’s Methylene Blue Solution (Merck, Darmstadt, Germany) diluted with 0.9% saline in a 1:3 ratio was injected subserosal at each of four sites at a distance of 1.5 to 2.0 cm from the tumor. This is a modification of our established method [27] with a replacement of the ink with methylene blue. After the subserosal injection, the depots were gently massaged to enhance the lymphatic flow. Blue stained LNs were removed immediately and stored in separate histology cassettes on ice, and their SLNs were classified.

**Figure 1:**
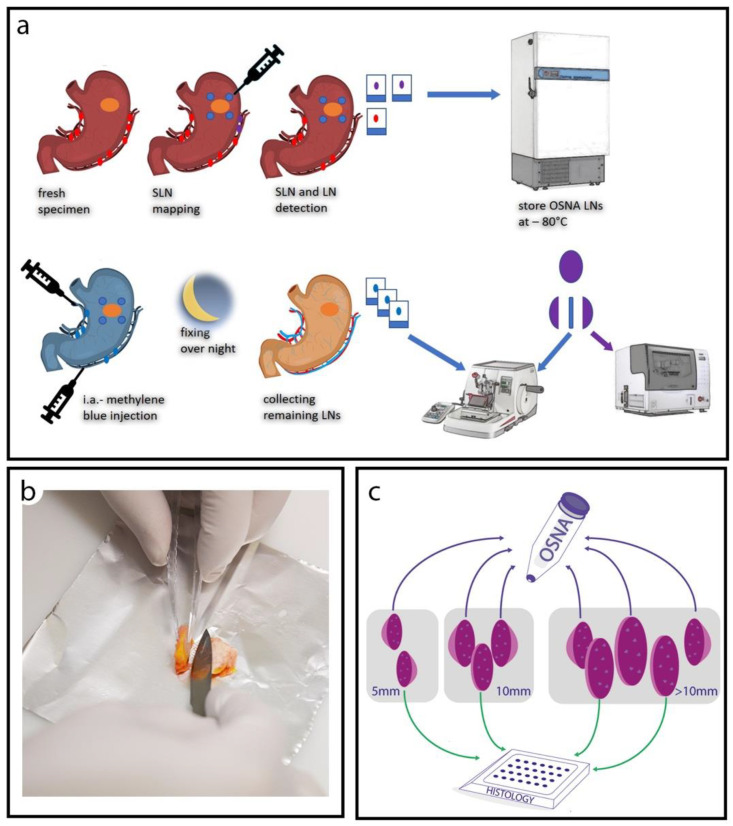
Tissue processing. (**a**) Specimen processing with SLN mapping and dissection of lymph nodes in the fresh state for mRNA-analysis. After completion, intra-arterial injection of methylene blue solution. After fixing LNs overnight, a second dissection of lymph nodes for the histological workup is performed. (**b**) Slicing of a frozen lymph node for the distribution between molecular and histological evaluation. (**c**) Scheme of tissue partitioning: small LNs of up to 5 mm short diameter were bisected, and half of the node was processed for histological evaluation, while the remaining half was used for OSNA analysis. In intermediated-sized LNs, between 5 to 10 mm, a middle slice of about 2 mm thickness was cut out for the histology, and the remaining parts of the node processed by OSNA. In large LNs (>10 mm), at least two slices were cut out for histology, while the rest was analyzed by OSNA.

Subsequently, the specimens were investigated for further non-SLNs. Each additionally identified LN was stored in a separate cassette on ice. Finally, all LNs were stored in a freezer at −80 °C. This procedure was limited to 45 min. After 1 to 5 days, the cassettes with the LNs were removed from the freezer. Each LN was individually measured and weighed. For the histology work up, samples from every LN were obtained and fixed in a 10% formalin buffer and embedded in paraffin (Figure 1a). Small LNs (<5 mm in short diameter) were bisected, and half of the node was processed for histological evaluation while the remaining half was used for OSNA analysis. For intermediate-sized LNs (5–10 mm), a middle slice of about 2 mm thickness was cut out for the histology, and the remaining parts of the node were processed by OSNA. In large LNs (>10 mm), at least two slices were cut out for histology, and the rest were analyzed by OSNA. To avoid RNA contamination, each LN was placed on a new piece of clean aluminum foil using new disposable instruments. The samples were kept cool throughout the preparation process. The tissue samples for the OSNA analysis were cut into smaller pieces, inserted into 1.5-mL reaction tubes, and stored at −80 °C (Figure 1b,c).

### 2.3. Immunohistochemistry

CK19 staining (Clone A53-B/A2.26, 1:100; Cell Marque, Rocklin, CA, USA) was performed on a Ventana Roche benchmark platform (Roche Diagnostics, Mannheim, Germany). The CC1 system was used with heat pretreatment for the antigen retrieval, and the reactions were developed using the Optiview detection system (Roche Diagnostics). External positive and negative controls were included in each run.

### 2.4. OSNA

**Figure 2:**
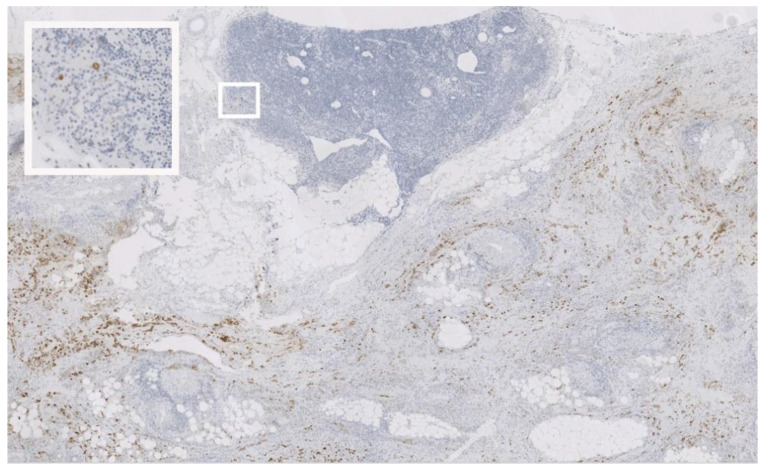
CK19-immunohistochemistry; Original magnification: 5×; A lymph node with surrounding fatty tissue can be appreciated. CK19-positive cells are found almost exclusively outside the lymph node. Only a few isolated tumor cells are identified—(insert: magnification 40×). This lymph node was positive by OSNA.

The OSNA analysis was performed using the Sysmex RD-100i system (Sysmex Europe, Norderstedt, Germany). Preparation was done according to the manufacturer’s instructions. The LN samples were homogenized with a LYNORHAG lysis buffer for 1.5 min, followed by centrifugation with 10,000× *g* for 1 min. Then, the LYNOAMP BC gene amplification reagent was added to the supernatant. CK19 mRNA was detected by the RT-LAMP method [28]. A cutoff of 250 CK19 copies/µL was used for differentiating between negative and metastatic LNs. Samples in which no LN structure could be confirmed histologically were excluded from the data analyses. Moreover, one positive OSNA result was excluded because histology and immunohistochemistry revealed a mainly dense peri-nodal infiltrate and only a few isolated tumor cells in the LN (Figure 2).

### 2.5. Statistics

Mean values were calculated with ±1 standard deviation. For the comparison of normally distributed continuous data, the double-sided Student’s *t*-test was used. Categorized data were analyzed with Fisher’s exact test. Correlations were calculated using the Spearman rank-order correlation coefficient. A *p* value < 0.05 was considered significant.

## 3. Results

### 3.1. LN Retrieval

**Figure 3:**
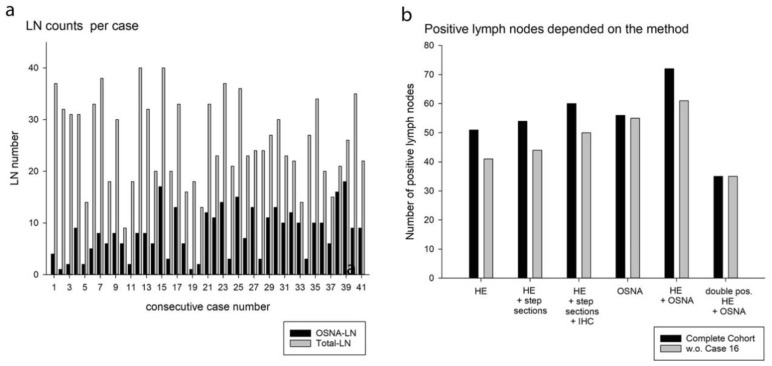
Lymph node yield and number of positive lymph nodes. (**a**) Number of identified lymph nodes in fresh state and after formalin fixing; the whole number of analyzed OSNA lymph nodes is 334; in total 1060 have been evaluated. (**b**) Identified positive lymph nodes depended on the method.

All LN dissections were performed by one experienced pathologist (BM). The total number of retrieved LNs, including LNs in the fresh state, was 1060, with a mean number of 26 ± 8 LNs (median: 24; range: 9–40). An additional 174 LNs were retrieved separately, completing the dissection of the D2 compartment, but were not included in further analysis in this study. LN detection in the fresh state for the OSNA RNA-based analyses was successful in all but one case. The total number of unfixed LNs was 334 with a mean yield of 8 ± 5 LNs (median: 8; range: 1–18), but this differed considerably between the cases (range: 1–18). The mean number of freshly sampled LNs differed significantly between the first and second study period with 6 ± 4 (median: 6; range: 1–6) vs. 10 ± 4 LNs (median: 10; range: 3–10) (*p* < 0.001) (Figure 3a). These LNs represented 31% of all identified local LNs. Again, this portion increased during the study, with mean values of 23% vs. 42% in the first and second period, respectively. The time for dissecting LNs in the fresh state was measured in the first eight cases. On average, it took 37 ± 7 (median: range: 25–50) minutes to dissect the LNs. After that, LN dissection on the fresh specimens lasted no longer than 45 min total.

### 3.2. SLN Mapping

SLN mapping was intended in 40 of 41 cases. In one case, SLN mapping was not performed because the specimen was already opened. SLN detection was successful in 29 cases. A total of 61 SLNs were detected, with a mean number of 2 ± 1 SLNs (median: 2 range: 1–6) per case. The final LN status based on conventional histological analysis was predicted correctly in 23 out of these 29 cases (79%), with 11 node-positive and 12 negative cases. Positivity in this context was defined as metastasis detection regardless of the method. In six cases, the SLNs were false negative. In one of these cases, all LNs were histologically negative for metastases, while one non-SLN was positive by OSNA analysis only. The other five cases showed advanced LN involvement (pN2/3). All six cases were treated by neoadjuvant chemotherapy. Involvement of the lymph vessel (L1) was found in three cases (50%). The success rate parameters for the SLN mapping are given in Table 2.

LN metastasis: LN status based on conventional histology and immunohistochemistry.

The conventional histopathological workup included H&E staining with step sectioning and was facilitated by immunohistochemistry of all conventionally retrieved, negative LNs from the gastric specimens (OSNA portion and secondary dissection). For the conventional histology, 18 out of 41 cases (44%) were node positive, with a mean number of 5 ± 6 (median: 5; range: 1–24) positive LNs per case. The total number of positive LNs was 97.

In one of these 18 cases (5%), the single metastasized LN was not included in the portion of LNs dissected in the fresh state (OSNA portion). Discrimination between LN-positive and negative cases was possible in 98% of all cases by evaluating the 334 LNs of the OSNA portion. In this portion, a total of 51 LN metastases were detected by initial H&E staining, with a mean of 3 ± 3 LNs (median: 2; range: 1–10) per positive case. In five cases, CK19 immunohistochemistry identified six additional positive LNs, four of which had micro-metastases and two of which had macro-metastases. The additional positive LNs did not cause upstaging from N0 to N+. An additional eight LNs showed isolated tumor cells. According to the current TNM classification, these were classified as N0. In three LNs from three cases, the metastases (size: 1.0–1.5 mm) were only detected in the subsequent step sections. In one of these cases, this was the only conventionally detected metastasis relevant for classification as N+.

### 3.3. OSNA LN Evaluation

The OSNA LN evaluation allowed for correct discrimination between node-positive and negative cases in 40 out of 41 cases (98%), corresponding to a sensitivity of 94.4%; a specificity of 100.0%; and negative and positive predictive values of 95.8% and 100.0%, respectively. In the single false-negative case, the only positive LN was not included in the OSNA portion of the retrieved LNs and thus could not be analyzed by OSNA. The overall LN positivity rate increased by three cases (from 18 to 21) due to the detection of single metastases by OSNA LN evaluation only. This corresponded to an upstaging rate of 14%. The LN positivity rate increased from 44% (19 cases) to 54% (22 cases). OSNA detected 55 positive LNs. The mean CK19 RNA copy numbers per LN were 92,477 copies/µL, and the median numbers were 4550 ± 251,442 copies/µL. Discrepancies between histological and RNA-based evaluation occurred in a total of 35 LNs (11%) and in 20 cases (49%).

Regarding the final SLN status, OSNA showed a non-significant trend (*p* = 0.311) towards a higher chance of predicting the final SLN status (10 out of 11; 90%) compared to initial H&E staining (7 out of 11; 63%). This corresponded to a sensitivity of 90.9%; a specificity of 100%; and negative and positive predictive values of 92.9% and 100.0%, respectively. The false-negative case by OSNA was a micro-metastasis.

In 16 LNs from five cases, the metastases were detected by histology only, while OSNA revealed no CK19 copies. Of these 16 LNs, 10 belonged to Case 16. Immunohistochemical staining against CK19 showed a strong expression in all 10 LNs, and therefore the reason for this false negativity remains unclear. One histologically negative LN in Case 16 showed positivity by OSNA and could therefore also be classified as N+ by OSNA. The mean LN size of OSNA SLN was 6 ± 4 mm (median: 6; range: 1–35 mm).

For 19 LNs that were initially determined to be negative by H&E histology with step sectioning, 15 cases were later revealed to be positive in the OSNA-based analysis. One evaluation was excluded from the analysis because of an extraordinarily high copy number level in a 4-mm-sized histological and immunohistochemical negative LN. In six of these LNs, CK19 immunohistochemistry revealed metastatic involvement (one diffuse infiltration, one macro-metastasis of 3 mm, and four micro-metastases). The mean copy number in the 19 H&E-negative, OSNA-positive cases was 3497 ± 6432 copies/µL (median: 940; range: 260–28,000 copies/µL).

**Figure 4:**
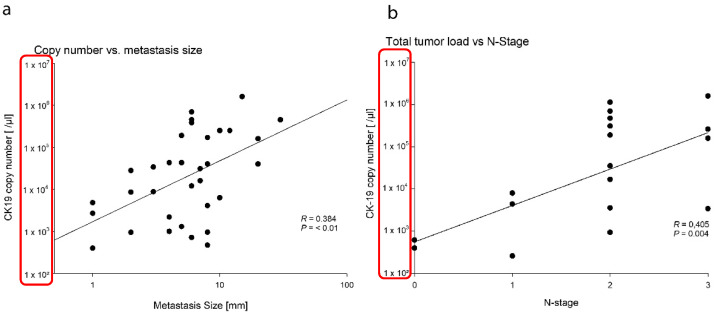
(**a**) Correlation between copy number/µL and matastasis diameter. (**b**) Correlation between total tumor load and N-stage.

The results of the metastasis detection by OSNA and the non-RNA methods are summarized in Figure 3b. The histologically measured maximum diameter metastases size correlated weakly but significantly with the CK19 copy number (*R* = 0.384, *p* < 0.01) (Figure 4a). The total tumor load (TTL; cumulative CK19 copy number per case) showed a weak but significant correlation with the final N stage (*R* = 0.405, *p* = 0.004) (Figure 4b). The sensitivity, specificity, negative predictive value, positive predictive value, and concordance are given in Table 2.

### 3.4. OSNA Upstaged Cases

In three male patients between 55 and 84 years of age, metastases were only detected by OSNA. The copy number values were low in all cases, ranging between 400 and 620 copies/µL. The classifications of the three G2 tumors were pT1b, 2, and 3. No neoadjuvant therapy was applied. The eldest patient with a pT2 tumor developed distant metastases and died 18 months after initial surgical therapy. Within the follow-up times of 29 and 38 months, no tumor progression occurred in the other two patients. The progression rate in the cases where node-negativity was also confirmed by OSNA evaluation was considerably lower with two out of 20 cases (10%, not significant).

## 4. Discussion

To the best of our best knowledge, this is the first study outside Japan to apply the OSNA technique for LN analysis in gastric cancer. The high efficacy of OSNA in detecting LN metastases has been shown in former studies from Japan (Table 3) [22,23,24,25]. Therefore, it was the aim of this study to evaluate OSNA in a European center, where it is unusual to perform post-operative LN dissection in the unfixed state. Moreover, we aimed to address whether it is possible to improve the overall sensitivity by integrating OSNA and ex vivo SLN mapping into the LN workup for gastric cancer.

In contrast to Japan, in Western countries it is not surgeons but pathologists who are responsible for dissecting LNs from organ specimens (with the exception of SLN mapping). Because it is technically easier, pathologists prefer to dissect LNs after fixing them overnight. Comparing the first and second study periods, we found a significant increase in the primary LN yield in terms of the number and proportion of all detected LNs (6 ± 4 vs. 10 ± 4 LNs; 23% vs. 42%) in the second period. This indicates a learning curve that has been traversed. It is important to emphasize that all LNs were dissected by the same pathologist, as this gives an idea of how many cases are necessary to achieve a certain level of routine.

Compared to a previous study involving some of the same authors [27], the ex vivo SLN mapping results were poor (Table 2) in this study, with detection rates of 73% vs. 87% and an accuracy of 58% vs. 93%. A low detection rate was the main factor for the low accuracy and was most likely caused by a modification of the method; the method was modified because of the concern that ink—used as a tracer in the previous study—could influence the RT-LAMP technique. Moreover, in the current study, SLNs had to be identified in the unfixed state. While the original protocol achieved results that were comparable with the favorable results of Japanese authors using in-vivo SLN mapping [23,25], this was not true in the current study. The efficacy of the protocol was too low to enable restricting the analysis to the identified SLNs. Therefore, the protocol requires improvement. Changing the tracer could be a reasonable approach because substances like indocyanine green, in combination with an ultrared ray detection system [8] or new magnetic tracers [29], have been shown to be very effective. These methods are more expensive compared to conventional dyes; however, the costs for the analysis of more than two samples with OSNA are considerably higher. Rakislova et al. [30] chose a different cost-saving approach to OSNA for colorectal cancer testing by pooling LNs up to a limit of 600 mg per microtube. An improvement in the hardware now available solves the limitation of the instrument we are still using to process only four samples at a time, which at least allows for more efficient use of personnel.

The OSNA technique was applied successfully in all but one case. In this case, 10 LN metastases could be readily identified by histology but were negative according to OSNA. Additionally, the single positive LN by OSNA was negative in the histology workup. Because of a strong CK19 expression in all of these false-negative LNs by immunohistochemistry, the reason for this failure remained unclear even after discussion with the manufacturer’s specialists. A processing error, therefore, remains the most plausible cause. Excluding this single case, the results are comparable with previous studies (Table 3) [22,23,24,25], with a high concordance of 92.4%.

Moreover, the overall LN positivity could be correctly assessed in all but one case in which the single positive LN was not part of the OSNA portion but was dissected after formalin fixing. Three cases turned out to be LN positive after OSNA evaluation. The use of immunohistochemistry also tended to increase the number of metastases identified but did not result in upstaging from N0 to N+ in this study. Out of the three OSNA upstaged cases, one showed tumor progression (33%). In comparison, the rate in the final node-negative cases without CK19 mRNA detection was only 10%. Due to these low numbers, these results were not significant and could have been a random effect.

In this context, it is essential to emphasize that the comparison of the methods with regard to their effectiveness in detecting metastases is limited. This is because for the various modalities, different tissue fractions were inevitably supplied, and the occasional occurrence of sampling errors must be taken into account. Because this study and previous ones have documented the efficacy of OSNA, the question of whether it would be more appropriate to dispense with the histological examination altogether arises. We recommend confirming that the sample used for OSNA is indeed part of the LN, and in the case of CK19 mRNA detection, this is not just a peri-nodal tumor manifestation without relevant node involvement. Notably, in gastric cancer of the diffuse type, infiltration beyond the muscularis propria is often only detectable by microscopy. We strongly believe that OSNA has to be combined with a histological step confirming the existence of a lymph node in the sample and ruling out only peri-nodal tumor infiltration (see Figure 2).

In this study, the metastases’ size correlated with CK19 copy number/µL. This is in concordance with the study by Yaguchi et al. [24], although their reported correlation was considerably stronger. TTL, which was used by Peg et al. [31] for detecting breast cancer, is defined by the cumulative CK19 copy number of all affected LNs in an individual case. In this study, TTL correlated with the N stage and showed the potential to be a count number-dependent prognostic method.

As mentioned above, the system-immanent problem that sample errors cannot be excluded because the samples have to be divided was a relevant limitation of this study. A further limitation was the number of cases, which was appropriate for investigating the effectiveness on an LN basis but was not high enough to give a definitive answer as to whether OSNA is clinically superior over conventional LN staging regarding the clinical outcome. A further limitation was that some of the D2-lymph nodes could not be integrated because of their separate dissection during surgery. It also has to stated that the inclusion of advanced stages and neoadjuvantly treated cases have an influence on the lymph nodes stage and are not optimal for the evaluation of the prognostic value of this technique. The careful investigation of all primary negative LNs by step sections and immunohistochemistry, as well as the provision of follow-up data, were strengths of this study.

## 5. Conclusions

In conclusion, the chosen ex vivo sentinel mapping concept failed to provide an effective method for selecting LNs and needs to be improved because the higher costs for applying the OSNA technique in cases with several LNs would be a significant obstacle to transferring the technology into the routine. However, this study showed that LN staging by the OSNA technique in gastric cancer was immediately feasible in a European center. Moreover, compared to conventional histology, upstaging from N0 to N+ occurred in three cases. The combination of conventional histology and OSNA revealed the highest number of node-positive cases. The clinical significance of these additionally identified cases needs to be evaluated in larger studies.

## Figures and Tables

**Table 1:** Clinicopathological data.

Parameter	Number (%)
N	41
Mean Age [years]	65 ± 13
Age < 50 years	6 (15%)
Gender [m:f]	1:0.41
pT0	6 (15%)
pT1	10 (24%)
pT2	4 (10%)
pT3	19 (46%)
pT4	2 (5%)
pN+	18 (44%)
Mean LN count	26 ± 8
L1	11 (27%)
V1	3 (7%)
c/pM1	8 (20%)
UICC stage IA	7 (17%)
UICC stage IB	3 (7%)
UICC stage IIA	5 (12%)
UICC stage IIB	7 (17%)
UICC stage IIIA	4 (10%)
UICC stage IIIB	1 (2%)
UICC stage IV	8 (20%)
Intestinal Typ Lauren	24 (59%)
Diffuse Typ Lauren	6 (15%)
Mixed Typ Lauren	8 (20%)
G1/2 *	16 (39%)
G3 *	23 (56%)
Neoadjuvant therapy	21 (51%)

* not available for two specimens after resection.

**Table 2:** Diagnostic value parameters of OSNA.

Author	Year	LNs	Cases	SN	T-Stages	Sensitivity	Specificity	Concordance	PPV	NPV	Study Objective
Yaguchi et al. [24]	2011	162	32	No	T1-T4	88.9	96.6	94.4	90.9	95.8	Evaluate optimal mRNA marker for OSNA and its efficacy in a clinical setting
Kumagai et al. [22]	2012	394	61	No	T1-T4	83.3	95.9	94.2	76.3	97.3	Multicenter study to evaluate OSNA in detecting metastases
Shimada et al. [23]	2019	439	43	Yes	T1 *	63.6 **	98.8 **	97.0 **	73.7 **	98.1 **	Comparison of OSNA in the setting of SN in early cancer
Shoji et al. [25]	2019	48	20	Yes	T1	NA	NA	NA	NA	NA	Evaluate in the setting of SN mapping by single-tracer imaging
Märkl et al. [27]	2020	334	41	Yes ***	T1-T4	68.6/85.4 ^#^	93.3/93.5 ^#^	89.5/92.4 ^#^	64.8/66.0 ^#^	94.3/93.5 ^#^	Ultrastaging

^#^ after exclusion of one case with 10 false-negative LNs by OSNA due to a technical problem; * study was planned for early cancers; 6 cases turned out to T2 or T3 (3 each); ** compared to permanent histology; PPV and NPV calculated from the published data; *** ex-vivo-SLN.

**Table 3:** Sentinel mapping efficacy parameters.

Parameter	Formula	%
Detection rate (%)	$\frac{\mathrm{Number}\;\mathrm{of}\;\mathrm{patients}\;\mathrm{with}\;\mathrm{successfully}\;\mathrm{retrieved}\;\mathrm{SLN}\;\_\;100}{\mathrm{Number}\;\mathrm{of}\;\mathrm{patients}\;\mathrm{enrolled}}$	73
Sensitivity (%)	$\frac{\mathrm{Number}\;\mathrm{of}\;\mathrm{patients}\;\mathrm{with}\;a\;\mathrm{tumor}\;\mathrm{involved}\;\mathrm{SLN}\;\_\;100}{\mathrm{Number}\;\mathrm{of}\;\mathrm{patients}\;\mathrm{with}\;\mathrm{metastases}\;\mathrm{in}\;\mathrm{any}\;\mathrm{lymph}\;\mathrm{node}}$	58
False negative rate (%)	100%-sensitivity	42
Accuracy (%)	$\frac{\mathrm{Number}\;\mathrm{of}\;\mathrm{patients}\;\mathrm{with}\;\mathrm{correct}\;\mathrm{prediction}\;\mathrm{of}\;\mathrm{the}\;\mathrm{nodal}\;\mathrm{status}\;\_\;100}{\mathrm{Number}\;\mathrm{of}\;\mathrm{patients}\;\mathrm{enrolled}}$	58

## Data Availability

The data presented in this study are available on request from the corresponding author.
